# Bacteriophage populations mirror those of bacterial pathogens at sites of infection

**DOI:** 10.1128/msystems.00497-23

**Published:** 2023-08-01

**Authors:** N. L. Haddock, L. J. Barkal, P. L. Bollyky

**Affiliations:** 1 Immunology Program, School of Medicine, Stanford University, Stanford, California, USA; 2 Division of Pulmonary, Allergy, and Critical Care Medicine, School of Medicine, Stanford University, Stanford, California, USA; 3 Division of Infectious Diseases and Geographic Medicine, School of Medicine, Stanford University, Stanford, California, USA; Quadram Institute Bioscience, Norwich, Norfolk, United Kingdom

**Keywords:** phage, bacteriophage, infection, bacterial infection

## Abstract

Bacteriophages, viruses that parasitize bacteria, are known to be abundant at sites of bacterial colonization, but the relationship between phages and bacteria at sites of infection is unclear. Bacteriophages are highly specific to their bacterial host species, and so we hypothesize that phage populations would mirror those of bacterial pathogens within infected tissues. To test this, here we study publicly available cell-free DNA (cfDNA) generated using next-generation sequencing of infected bodily fluids, including urine, joint fluid, peritoneal fluid, bronchoalveolar lavage fluid, cerebrospinal fluid, and abscess fluid, as well as uninfected control samples. These were analyzed using a computational pipeline for identifying bacteriophage sequences in cfDNA. We find that bacteriophage sequences are present in both infected and uninfected bodily fluids and represent a variety of bacteriophage morphologies and bacterial hosts. Additionally, phages from *Escherichia coli*, *Streptococcus*, and *Staphylococcus aureus* are overrepresented both in terms of proportion and diversity in fluids infected with these same pathogens. These data indicate that phages reflect the relative abundance of their bacterial hosts at sites of infection. Bacteriophage sequences may help inform future investigative and diagnostic approaches that utilize cell-free DNA to study the microbiome within infected tissues.

**IMPORTANCE:**

Bacteriophages are an active area of investigation in microbiome research, but most studies have focused on phage populations at sites of bacterial colonization. Little is known about bacteriophage ecology at sites of active infection. To address this gap in knowledge, we utilized a publicly available data set to study bacteriophage populations in cell-free DNA collected from sites of infection. We find that phages reflect the relative abundance of their bacterial hosts at sites of infection. These studies may lead to future investigative and diagnostic approaches that incorporate phages as well as bacterial cell-free DNA.

## OBSERVATION

Bacterial infections are a major public health issue, causing morbidity and mortality worldwide. Recent studies indicate that bacterial infections may cause 13.6% of all global deaths (1). In recent decades, the rise of antimicrobial resistance in bacterial infections has become increasingly common (2) and poses a growing threat to vulnerable populations. The study of bacterial ecology at these sites is critical for understanding infection outcomes and developing improved diagnostics and therapeutics. However, much remains unknown.

Bacteriophage (phage) are viruses which infect bacteria, and they are highly specific to their bacterial hosts (3). Phage are present ubiquitously throughout the human body (4, 5), and both reflect and influence bacterial populations. For this reason, phage are an attractive therapeutic target (6, 7), but outside of the study of phage therapy, endogenous phages have been largely disregarded. Phage have been shown to be associated with bacterial host characteristics, such as antimicrobial resistance (8) or infection chronicity (9). Little is known about phage ecology in the human body as a whole, but what is known is mostly relevant to sites of bacterial colonization, such as in the gut (5, 10). Aside from recent work describing phage populations in chronically infected tissues (11), the relationship between phages and bacteria at sites of infection is mostly unclear.

Here, we have investigated phage populations in infected bodily fluids and compared these versus uninfected controls. To accomplish this, we utilize next-generation sequencing data of cell-free DNA (cfDNA) from a publicly available study. cfDNA are short unencapsulated DNA sequences and can be found in circulation in plasma as well as in bodily fluids. Though being largely comprised human sequences, reflects microbial—and bacteriophage—sequences (12). They are, therefore, a strong candidate for analysis of microbial ecology in the context of infection. We utilize here a publicly available data set of 76 infected bodily fluid samples (Bioproject PRJNA558701) generated via Illumina sequencing (13). These included samples from infected wounds, joints, urine, serum, and other sites as well as culture negative controls. Causative infectious agents were identified by culture or 16S rRNA sequencing, including *Escherichia coli*, *Streptococcus* spp., and *Staphylococcus* spp. This data set provides an excellent setting to investigate the relationships between pathogen and bacteriophage at the site of infection.

**Fig 1:**
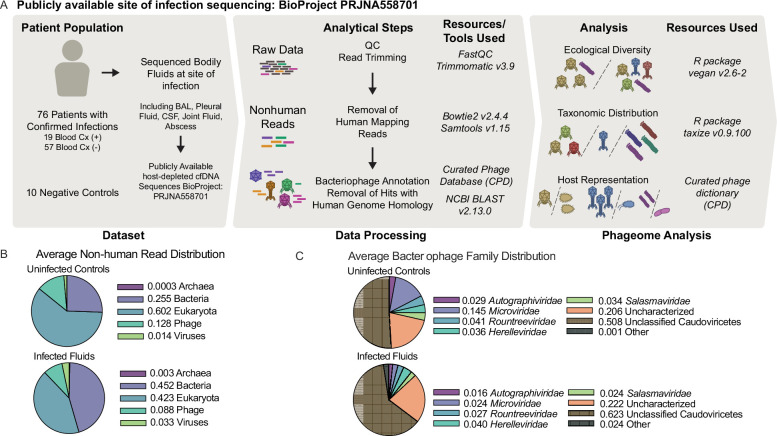
Infected and uninfected bodily fluids possess bacteriophage sequences. (**A**) Schematic of sample numbers, sample processing, and analytical approach. (**B**) Distribution of non-human reads identity by Archaeal, Eukaryotic, Bacterial, Mammalian Viral, and Bacteriophage categories. For uninfected controls, Archaea: mean 0.0003, SD 0.0005; Eukaryotic: mean 0.602, SD 0.214; Bacterial: mean 0.255, SD 0.238; Mammalian Viral: mean 0.014, SD 0.017; and Bacteriophage: mean 0.128, SD 0.102. For infected fluids, Archaea: mean 0.003, SD 0.028; Eukaryotic: mean 0.423, SD 0.330; Bacterial: mean 0.452, SD 0.370; Mammalian Viral: mean 0.033, SD 0.073; and Bacteriophage: mean 0.088, SD 0.111. (C) Average bacteriophage distribution by bacteriophage family.

To identify phages within the metagenomic sequencing data, we apply a previously described bacteriophage annotation pipeline (Fig. 1A) (14). In brief, raw data were quality controlled and trimmed, human host reads were subtracted by mapping to the human reference genome, and a BLAST search utilizing the full NCBI Nucleotide database was used to assess non-human cfDNA proportions. This revealed that on average, 8.8% of identifiable non-human reads were from bacteriophage in infected fluids, indicating that at the average site of infection, bacteriophage comprises a considerable proportion of free DNA (Fig. 1B). Of note, an average of 12.8% of non-human reads belongs to bacteriophage in the uninfected surgical control fluid samples—indicating that bacteriophage are abundant in these fluids independent of infection status. A first-pass BLAST search (15, 16) was performed as described with our previously described Curated Phage Database with stringent removal of sequences with human genome homology (14). To link phage with bacterial host(s), we utilize a Curated Phage Dictionary (14), naming convention of bacteriophages with clearly identified host genera, and NCBI nucleotide source host fields for bacteriophage sequence entries. This dictionary includes taxonomic classifications for both the phage and the bacterial host, if known. Subsequent annotations reflect a diverse pool of bacteriophage across many families (Fig. 1C).

**Fig 2:**
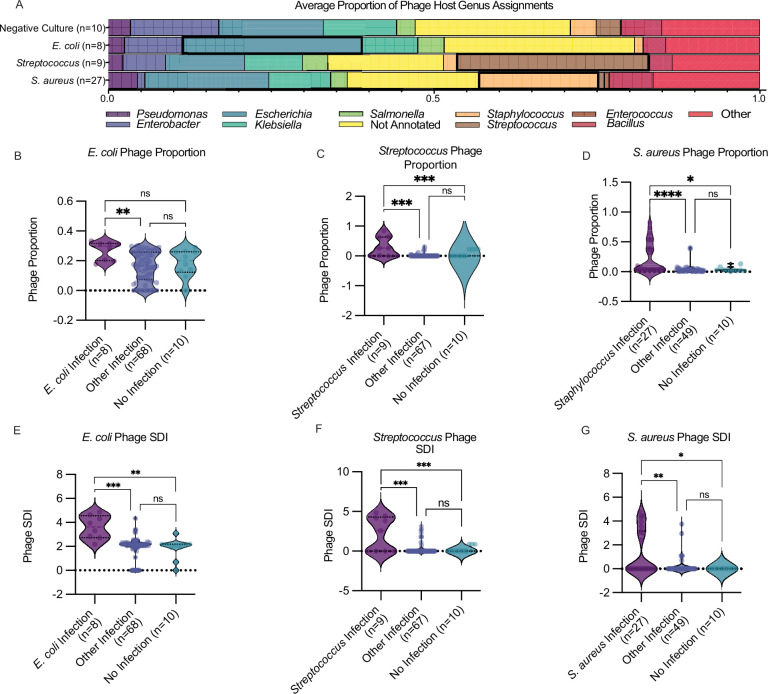
Local infections are associated with increased pathogen-associated phage proportion and diversity. (A) Average distribution of unique phages by bacterial host genus by infection category, with causative pathogen’s own bar outlined in black outline. (B–D) Infection-specific phage proportion calculated and shown as violin plots with median and quartiles shown by dashed lines for (B) *E. coli* (Kruskal-Wallis *P* = 0.0083, mean *E. coli* Infection = 0.2666, mean Other Infection = 0.1575, mean No Infection = 0.1842. Dunn’s Multiple comparisons: *E. coli* Infection vs Other Infection *P* = 0.0062, *E. coli* Infection vs No Infection *P* = 0.1456, and Other Infection vs No Infection *P* > 0.9999), (C) *Streptococcus* (Kruskal-Wallis *P* = 5.44E−5, mean *Streptococcus* Infection = 0.2939, mean Other Infection = 0.0145, and mean No Infection = 0.00. Dunn’s Multiple comparisons: *Streptococcus* Infection vs Other Infection *P* = 0.0001, *Streptococcus* Infection vs No Infection *P* = 0.0002, and Other Infection vs No Infection *P* = 0.8642), (D) *S. aureus* (Kruskal-Wallis *P* = 3.14E−5, mean *S. aureus* Infection = 0.2215, mean Other Infection = 0.0418, and mean No Infection = 0.0411. Dunn’s Multiple comparisons: *S. aureus* Infection vs Other Infection *P* = 1.98E−5, *S. aureus* Infection vs No Infection *P* = 0.0400, and Other Infection vs No Infection *P* > 0.9999), (E–G) Infection-specific SDI calculated and shown as violin plots with median and quartiles shown by dashed lines for (E) *E. coli* (Kruskal-Wallis *P* = 0.0003, mean *E. coli* Infection Phage SDI = 3.591, mean Other Infection Phage SDI = 1.884, and mean No Infection Phage SDI = 1.854. Dunn’s Multiple comparisons: *E. coli* Infection vs Other Infection *P* = 0.0002, *E. coli* Infection vs No Infection *P* = 0.0018, and Other Infection vs No Infection *P* > 0.9999), (F) *Streptococcus* (Kruskal-Wallis *P* = 0.0003, mean *Streptococcus* Infection Phage SDI = 2.187, mean Other Infection Phage SDI = 0.2422, and mean No Infection Phage SDI 0.00. Dunn’s Multiple comparisons: *Streptococcus* Infection vs other infection *P* = 0.0002, *Streptococcus* Infection vs No Infection *P* = 0.0003, and Other Infection vs No Infection *P* = 0.3549), (G) *S. aureus* (Kruskal-Wallis *P* = 0.0036, mean *S. aureus* Infection Phage SDI = 1.362, mean Other Infection Phage SDI = 0.2178, and mean No Infection Phage SDI 0.00. Dunn’s Multiple comparisons: *S. aureus* Infection vs other infection *P* = 0.0093, *S. aureus* Infection vs No Infection *P* = 0.0223, and Other Infection vs No Infection *P* > 0.9999).

We find that when comparing overall composition of phageomes by bacterial host—there is notable enrichment for pathogen-specific phage by infection etiology and that there is a non-zero phage background in the negative control samples which possessed *Escherichia*, *Klebsiella*, *Enterobacter*, and other phages (Fig. 2A). When analyzing these local phageomes with respect to infection etiology, we find that *E. coli* phage are increased in proportion compared to other infections (Fig. 2A and B). We find similar trends in *Streptococcus* and *Staphylococcus aureus* infections (Fig. 2A, C, and D). Ecological diversity of phage, as calculated by the Shannon Diversity Index (SDI), a measure of entropy often applied in ecology to quantify species richness and evenness (17), is disrupted on a per-infection basis (Fig. 2E through G), with higher levels of specific phage diversity in patients with infection by the corresponding host but not in controls or those with other infection etiology.

Taken together, these findings demonstrate that bacteriophage populations reflect bacterial pathogens in infected bodily fluids.

However, this work possesses several limitations. It is unclear whether the sequences characterized here are from active bacteriophage particles, free phage DNA, or prophage DNA from bacterial hosts—in part due to the relatively low depth of sequencing of non-human cfDNA in these samples preventing generation of phage contigs. Further studies are needed to understand the sources of these DNA as well as how they enter these bodily fluids. Another limitation is that this study utilizes publicly available cfDNA data taken from one patient cohort and may reflect geographically enriched features. Furthermore, the number of uninfected controls is low (10) in comparison to a number of infected fluids (76), and it is possible that this data set is underpowered for the detection of signals distinguishing uninfected from infected fluids beyond infection-specific enrichment of phage proportion and diversity. The number of samples additionally limits comparisons by the type of biosample, and future work should include a larger cohort for comparisons across different bodily fluids. Finally, the phage dictionary utilized here relies on characterized and sequenced phage genomes, which necessarily enriches for phages associated with human infections—it is possible that many environmentally associated phages are not being identified here due to a relative underrepresentation in sequence repositories.

In summary, we find that bacteriophage sequences are present in both infected and uninfected bodily fluids and represent a variety of bacteriophage morphologies and bacterial hosts. Additionally, we demonstrate that infection etiology is reflected in infected bodily fluids through pathogen-associated phage proportion and diversity. Bacteriophage sequences may help inform future investigative and diagnostic approaches that utilize cell-free DNA to study the microbiome within infected tissues.
